# Health Economic Challenge: Geriatric Trauma—An Age-Based Observational Analysis of Treatment Costs and Reimbursement Issues

**DOI:** 10.3390/ijerph19148270

**Published:** 2022-07-06

**Authors:** Cora Rebecca Schindler, Mathias Woschek, Nils Mühlenfeld, Lukas Seifert, Ingo Marzi, Philipp Störmann, René Danilo Verboket

**Affiliations:** 1 Klinik für Unfall-, Hand- und Wiederherstellungschirurgie, Goethe Universität Frankfurt, 60590 Frankfurt am Main, Germany; mathias.woschek@kgu.de (M.W.); nils.muehlenfeld@kgu.de (N.M.); marzi@trauma.uni-frankfurt.de (I.M.); philipp.stoermann@kgu.de (P.S.); rene.verboket@kgu.de (R.D.V.); 2 Klinik für Mund-, Kiefer und Plastische Gesichtschirurgie, Goethe Universität Frankfurt, 60590 Frankfurt am Main, Germany; lukasbenedikt.seifert@kgu.de

**Keywords:** mTBI, geriatric trauma, fall, DRG, reimbursement, health economics, treatment costs

## Abstract

Demographic change is having a major impact on the economic and structural development of the healthcare system. People stay active longer and the number of mild traumatic brain injury [mTBI] in patients ≥ 65 years of age consequently increases. The aim of this comparative analysis is to illustrate the impact of demographic change and the increasing treatment of geriatric trauma patients on the cost structure of the health care system using mTBI as an example diagnosis. Patients and Methods: The 12-month retrospective analysis included 220 in-patients treated with mTBI and remunerated under the German Diagnosis Related Group [G-DRG] B80Z. For comparative analysis, the patient population was divided into two study groups according to age [U65 18–64 years, G65 ≥ 65 years]. For the cost and proceeds calculation, itemized cost reports (personnel, supply, material, and equipment costs, etc.) were created. Results: 163 patients U65 and 57 patients G65 were included. In the G65 group, the most frequent accident mechanism was a fall from a short distance (84.1 vs. U65 36.7%; *p* = 0.007). For the inpatient admission of G65, the use of anticoagulants (*p* < 0.001) and comorbidity (*p* = 0.002) played a primary role, while for younger patients it was more neurological symptoms (*p* < 0.001) and alcohol (*p* < 0.001) that led to inpatient monitoring. The mean length of hospitalization of G65 patients was significantly longer than that of younger patients (G65 2.4 ± 1.9 days > U65 1.7 ± 0.8 days; *p* = 0.007) and radiological examinations (G65 94.7% > U65 23.3%; *p* = 0.013) were performed more frequently. Comparing analysis of the cost and proceeds of U65 vs. G65 results in a proceeds difference of €51,753.91 per year for the G-DRG B80Z compared to U65. Conclusions: It has been shown that there is a difference in costs and proceeds when comparing younger and older patients, resulting in a reimbursement deficit. In view of the demographic development in Europe, flat-rate remuneration will lead to a considerable discrepancy between DRG reimbursement and the coverage of hospitals’ running costs. Providing health care to an increasingly aging society represents one of the major personnel and financial challenges for the public health system in the coming decades. Further adaptation of the DRG system to the growing costs caused by older patients is imperative.

## 1. Introduction

Demographic change moving toward an aging society is having a major impact on the economic and structural development of the health care system. People are not only getting older but are fortunately remaining independent and active for longer [1]. As a result, the number of accidents in those of geriatric age ≥ 65 years is increasing. About 30–40% of this age group fall at least once a year; the common cause is a multifunctional gait disorder [2]. A relevant number of these patients suffer a mild traumatic brain injury [mTBI], which often leads to hospitalization, due to co-factors such as premedication with anticoagulants or multimorbidity [3,4]. In 2018, 284,462 patients (Germany) suffered a TBI with an average hospitalization of 4.0 days. Of these, 40% were geriatric patients who predominantly suffered a mild TBI. Extrapolated, the total public costs for TBI amount to about 2.8 billion euros per year [5]. For geriatric patients, rapid recovery is crucial, as mobility and independence are much more difficult to regain in older age but are fundamental in avoiding the need for long-term care [6]. Due to the advanced age at the time of trauma, indirect medical costs such as incapacity to work are lower in geriatric trauma patients compared to younger ones. However, longer hospitalizations, more complex treatments, later need for care and recurrent falls make the care of this patient group resource-intensive and poses a social, medical and health economic challenge [6,7].

Diagnosis Related Groups [DRG] have been used to manage the financing of health care since the 1980s. Due to their incentives and potential efficiency gains, DRG have been introduced across Europe. DRG refer to a classification system for a flat-rate billing procedure that groups hospital cases (patients) in a diagnosis-related case cluster with a similar cost structure [8]. Critics of the DRG system see a lack of adaptation to socio-demographic developments and a commercialization of the health care system that is associated with a significant increase in the workload of nurses and physicians [8,9].

Increasing demand for specialized healthcare accompanied by limited capacity has greatly increased interest in the analysis of disease-specific costs and reimbursement issues [10]. The objectives of this comparative analysis of geriatric (≥65 years) and younger trauma patients (<65 years), using the diagnosis of mTBI as an example, are twofold: (1) they illustrate the extent of the changing cost structure under the current demographic trend; and (2) they objectify the resulting economic consequences for the health care system.

## 2. Patients and Methods

### 2.1. Study Design

A one-time systematic query of the Hospital Information System (HIS) was used to identify all patients with mTBI (ICD-10 S06.0 [11]) treated in the Emergency Department [ED] of Goethe University Hospital, Frankfurt am Main, Germany, between 1 January 2019 and 31 December 2019. Of these, we included all patients (≥18 years) who were hospitalized and were counted under G-DRG B80Z. This is the per-case flat rate for head injuries, under which mild TBI is included for billing purposes [12]. The study contains only cases of patients with public health insurance. Patients with private health insurance or cases of occupational accidents were excluded due to different reimbursement modalities.

For this transversal observational study, the patient population was divided into two groups according to age:

- U65 = patients aged 18–64 years with mild TBI
- G65 = geriatric patients aged ≥65 years with mild TBI

Demographic and clinical parameters were collected by reviewing electronic medical records. The Identification of Seniors At Risk (ISAR) Score was assessed for all patients ≥65 years of age [13].

To calculate the treatment expenses, the medical and commercial controlling departments were consulted in the drafting of itemized cost lists (according to personnel, supply, material, and equipment costs, etc.). All tangible treatment costs, from the admission in the ED to subsequent inpatient treatment were included (Appendix A).

### 2.2. Statistical Methods

Means ± standard deviations [SD] were calculated for continuous, normally distributed variables. Categorical or continuous variables were mapped by medians with interquartile ranges [IQR]. The p-values for categorical variables were derived from Fisher’s two-tailed exact test. On continuous variables, the Mann-Whitney U test (Bonferroni post hoc test) was used. A *p* value < 0.05 was considered statistically significant. All analyses were performed using Statistical Package for Social Sciences (SPSS for Mac©), version 26 (SPSS Inc., Chicago, IL, USA), and graphs were created using GraphPad Prism 7 for Mac© (GraphPad Software Inc., San Diego, CA, USA).

## 3. Results

### 3.1. Demographics and Clinical Data of the Patient Collective

Within the 12-month study period, a total of 220 patients were hospitalized with mTBI (ICD-10 S06.0) and billed under the DRG B80Z. Of these, 163 patients were between 18 and 64 years of age [U65], and 57 patients were 65 years of age or older [G65].

**Figure 1:**
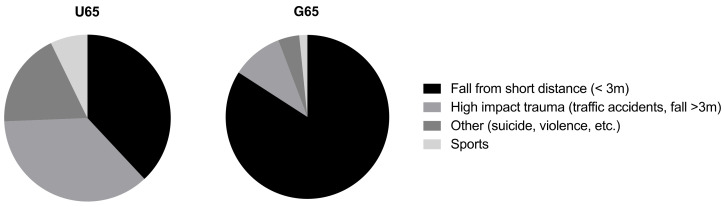
Accident mechanism of mTBI stratified by age U65 (n = 163) vs. G65 (n = 57). Fall from short distance (<3 m): U65 36.7 vs. G65 84.1% (*p* = 0.007). High impact trauma (traffic accidents, falls >3 m): U65 38.4 vs. G65 10.1%. Others, like violence or suicide: U65 18.6 vs. G65 4.2%. Sports: U65 7.3 vs. G65 1.7%.

The most common accident mechanisms of mTBI (Figure 1) in the U65 were high impact trauma (38.4%) and falls from a short distance (36.7%), followed by less common trauma mechanisms such as violence (18.6%) or sports (7.3%). In the G65, the most frequent cause of accidents was falls from a short distance (84.1 vs. U65 36.7%; *p*= 0.007). Other accident mechanisms played a minor role (between 1.7–10.1%).

Table 1 shows that the median age of the U65 was 40 (IQR 29–51) years and that of the G65 was 79 (IQR 74–86) years. The G65 achieved a median ISAR score of 4 (IQR 4–5) points. In the U65, about 10% more men than women were hospitalized with mTBI; while in the G65, gender made no difference.

The groups did not differ in their mean score on the Glasgow Coma Scale [GCS], but significantly more patients in the U65 group showed typical TBI neurological symptoms such as loss of consciousness, amnesia, or vomiting (U65 76.7 > G65 22.8%; *p* < 0.001).

As expected, significantly more patients ≥65 years of age had an oral anticoagulant in their pre-medication (*p* < 0.001), in addition to relevant pre-existing disorders (e.g., dementia or coagulopathy; *p* = 0.002). In contrast, an elevated blood alcohol level (≥0.3‰) was found significantly more often in younger patients (U65 20.9 > G65 12.3%; *p* < 0.001).

In 14% of the U65 and 33% of the G65 groups, a social indication such as lack of home care or living alone was the reason for inpatient admission.

In total, patients in this study spent 414 days (n = 220) in hospital with a mean length of stay of 1.9 (±1.4) days. By age, the mean length of hospitalization of G65 was significantly longer than that of younger patients (G65 2.4 ± 1.9 days > U65 1.7 ± 0.8 days; *p* = 0.007).

### 3.2. Length of Stay and Reimbursement Calculation

**Figure 2:**
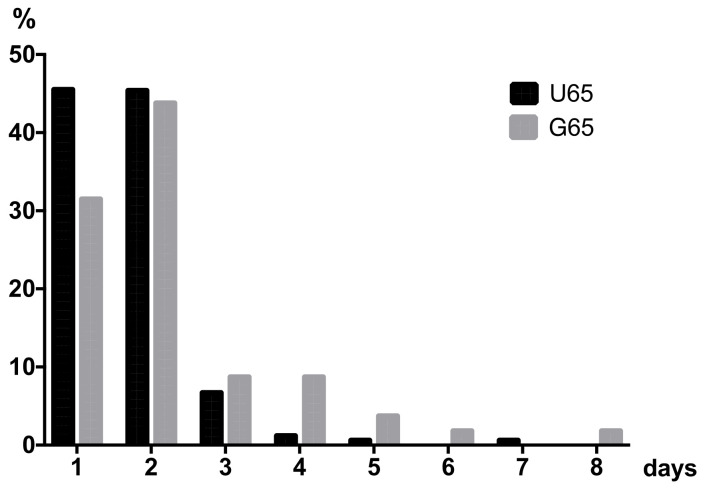
Length of stay [LOS] of patients with mTBI in 2019 in days (d) stratified by age U65 (n = 163) and G65 (n = 57).

Figure 2 shows that most patients (U65 90.8 vs. G65 75.4%) were observed in hospital within the first 24 to 48 h after trauma. One quarter (24.6%) of the G65 had an extended stay of up to eight days.

Based on the G-DRG B80Z, the reimbursement (Euro [€]) was calculated according to the number of cases in both groups (Appendix A).

In 2019, the total remuneration for mTBI (G-DRG B80Z) was €245,747.76. Of this, €174,043.69 was paid for U65 (n = 163) and €71,704.07 for the G65 (n = 57) groups. This results in an average revenue of €1067.75 per case in the U65 and €1257.97 per case in the G65.

### 3.3. Cost Calculation

The calculation of the costs for in-patient observation and monitoring in the case of mTBI was based on personnel, material and equipment costs as well as in-patient stay costs. A detailed calculation of personnel costs per minute can be found in Appendix B.

#### 3.3.1. Total Costs of Admission Treatment in the ED

In-house tariffs were used in the cost calculation for the diagnostics (Table 2) and the hospital stay. Cranial Computed Tomography [cCT] had the largest share of the costs for diagnostics (total €11,356.48). For G65 radiological examinations, cCT (G65 94.7 > U65 23.3%; *p* = 0.013) in particular was performed more frequently.

The calculation of total costs of admission treatment in the ED resulted in a total of €15,419.46 for U65 patients and an average cost of €94.60 per case. The total cost of emergency treatment for G65 was €12,268.62 with an average cost of €215.24 per case.

#### 3.3.2. Total Costs of Inpatient Treatment

After admission via ED, patients with mTBI stay on trauma wards or in a monitored bed, depending on the clinical condition and risk for secondary complications.

Total costs were calculated on the basis of inpatient days (Appendix B) within the respective age groups.

This resulted in total costs for clinical observation on the trauma ward of €68,459.61 for 214 inpatient days in the U65, which means €319.90 per day and € 539.05 per patient case. In the G65 group, the inpatient costs amounted to €26,999.01 for 76 inpatient days. This results in €355.25 per day and €843.72 per case.

Patients with mTBI spent a total of 124 days on a monitor bed because of medical requirements. The total costs for the 61 inpatient days of the U65 on a monitored bed amounted to €20,026.51, €328.30 per day results in €556.29 per case. The G65 group stayed 63 days to €14,052.24 on the monitor bed, which is €223.05 per day and €562.09 per case.

#### 3.3.3. Comparison of the Cost-Proceeds Calculation for U65 vs. G65

To calculate the actual cost-proceed ratio for all DRG B80Z cases in 2019, the calculated costs of the initial treatment in the ED as well as the costs for inpatient days (d) on the trauma ward (U65: 214 days, G65: 76 days) and in the monitoring bed (U65: 61 days, G65: 63 days) were added and subtracted from the reimbursement of DRG B80Z (Table 3).

The total costs for the U65 group amount to €103,905.58, which corresponds to €637.46 per case. This is offset by reimbursement from DRG B80Z amounting to €174,043.69, or an average of €1067.75 per U65 patient. After deduction of the costs incurred, €70,138.11 or an average of €430.30 per patient remain for the U65 group.

According to Table 3, DRG B80Z generated proceeds of €71,704.07 in the G65. Deducting the costs results in proceeds of €18,384.20 or an average of €322.53 per patient ≥ 65 years.

Comparing the cost and proceeds calculation of U65 to G65 it results in a proceeds difference of €51,753.91 per year for the G-DRG B80Z. In relation to the average case, the G65 thus generated on average €107.77 less proceeds per patient than the U65 group.

## 4. Discussion

The health care system is subject to increasing economization while society continues to age. The difficulties and consequences of the economization of the health care sector with relevant cost and proceeds deficits have already been addressed in publications as well as in the public discourse. Demographics are now posing additional challenges [14,15]. In this comparative analysis, it was shown for the first time that the treatment of geriatric trauma patients (≥65 years, G65, n = 57) with mild TBI (DRG B80Z [12]) leads to relevantly higher costs by higher diagnostic expense and resource consumption. Compared to the younger reference group (18–64 years, U65, n = 163) this leads to a relative proceeds difference of €107.77 per patient case and €51,753.91 per year for the diagnosis of mild TBI (ICD-10 S06.0).

### 4.1. Risk Factors in mTBI among Geriatric Patients: Anticoagulation and Co-Morbidity

Two hundred and twenty patients with mTBI were included in this study. The number of cases in the G65 (about one third of the total number of cases) shows that seniors, both men and women, here with a median age of 79 years, have accidents into old age. The G65 accounted for a quarter of all mTBI patients in 2019. With a median ISAR Score of 4, we assume a relevant geriatric character of this patient group, in which multifactorial gait instability plays a relevant role in the genesis of various injuries [2,6,13,16]. While younger patients fall from both high and low distances, as well as suffering equally from high-impact trauma, patients over 65 almost exclusively suffer mTBI after falls from a low height (less than three metres). For the admission of G65 patients, the use of anticoagulants (*p* < 0.001) and comorbidity (*p* = 0.002) played a significant role, while for younger patients it was more neurological symptoms (*p* < 0.001) and alcohol (*p* < 0.001) that led to inpatient observation. According to the German Society for Trauma Surgery, treatment of a mild TBI without relevant symptoms can often be done on an outpatient basis. The patient must be informed before discharge about the need to present again in case of loss of consciousness and the occurrence of new symptoms (nausea and vomiting) or a worsening of symptoms [17,18]. Under the influence of drugs and alcohol, neurological assessment and sanity are significantly impaired, so these cases are often admitted to hospital for monitoring [19,20]. Neurological symptoms were the secondary reason for admissions for those under 65 years of age. Older patients are less likely to show typical symptoms, partly due to reduced brain volume. Here, co-factors such as previous illnesses, prior medication or social indicators play a role. In these cases, admission for prevention and early detection of secondary complications is substantial [21]. The intake of anticoagulant medication is associated with a high risk of occult intracranial haemorrhages, i.e., without correlating symptoms. These may also occur hours after the trauma and may only then become apparent image-morphologically and clinically. In both cases, there can be severe disturbances of consciousness and even death [22]. It is therefore not surprising that cCT was performed significantly more often in those over 65 years of age compared to the younger group. Another reason for the difference is certainly radiation hygiene with restrained indication in younger patients [23].

### 4.2. Social Care Deficit Leads to Hospitalization

In 20% of the cases, the social situation of the patients played an important role in the indication for admission. This was more often the case for patients over 65 years of age, reflecting a serious problem of old age and one of the greatest challenges of demographic developments to the health care system. As people age, their ability to help themselves and care for themselves independently at home is compromised much more quickly than for those of a younger age. In addition, elders are often just at the limit of compensation living alone in their own households [24]. Often a “simple discharge” to home for these patients is difficult from an ethical and moral point of view.

### 4.3. G65: Higher Costs due to Longer In-Hospital Treatment

Patients with risk factors or symptoms usually require inpatient observation (with clinical monitoring of consciousness and pupillary motor function). They require one to three days of bed rest, after which symptoms usually resolve [25,26]. The prespecified length of stay for patients with mTBI was 2.2 days on average for G-DRG B80Z in 2019 [12]. In this study, the overall median length of stay was 1.9 days. It is noteworthy that 45% of U65s and 32% of U65s fell below the lower limit length of stay of two days and resulted in reductions in reimbursement. This reflects the impractical calculation of DRG B80Z. According to the current literature, various algorithms exist that usually require 6 to 24 h of observation, depending on the symptoms and the diagnostics performed [27]. Patients are often admitted at night or in the morning and discharged after 6, 12 or less than 24 h, often on the same calendar day, resulting in a reimbursement reduction of a factor of −0.142 per case or €503. Transferred to the year 2019 in this study, a deduction of €46,311 of total DRG B80Z results from falling below the lower limit of the prespecified length of stay.

The average reimbursement is approximately €190 higher for a G65 patient than for a U65 one. This is due to the additional inpatient days, which cost the health system a surcharge of 400 euros per day. The mean length of hospitalization of G65 was significantly longer than that of younger patients (*p* = 0.007), whereby the G65 more frequently exceeded the upper limit length of stay of three days. On the one hand, a longer inpatient stay may be necessary because of the diagnosis and treatment of the cause of trauma (e.g., syncope). On the other hand, elderly patients often take longer to recover from a fall and mTBI. Disruption of their daily routine and even short-term confinement to bed can lead to decompensation of their often already fragile socio-medical condition and make it difficult to return them to their home environment [7,24].

### 4.4. Challenge of Geriatric mTBI: Same Reimbursement but Higher Costs

It was shown that the treatment of older patients is associated with significantly higher costs for identical remuneration by DRG B80Z, resulting in less preceeds of around €108 per case compared to the U65 group, which is a total of €6142 in 2019, based on the total number of 57 patients. However, looking at clinical reality, the shortfall could be much larger. Forty five percent of the U65 group were below the predefined length of stay at discharge. If these were not subject to the above-mentioned deduction of reimbursement, the monetary gap between U65 and G65 would be much larger. The actual difference that arises between the groups is thus masked by the inflexible calculation of the DRG.

In addition to the calculable costs described above, there are cost factors that are difficult to capture. Inventory costs, including the cost of research and teaching at the University Hospital, special compensation for staff on night and weekend shifts, and other overhead costs are not part of the costing considered, which in reality would further reduce the surplus. Taheri, et al. describe this type of cost as a significant amount that often cannot be recovered through payment from the DRG system. Level-1 hospitals incur high costs because they must provide the maximum medical care available [28]. This contradicts the current trend towards smaller hospitals to minimize costs by cutting back on loss-making departments and excluding unprofitable services.

### 4.5. Adjustment of Reimbursement to Demographic Change Required

One possible solution is to consider adjusting the valuation scale for DRG B80Z. However, it should be noted that not all patients of every age can be billed the same, as this would not represent adequate remuneration. A simple differentiation according to the study groups U65/G65 and special risk groups is conceivable.

Another possibility would be the introduction of an additional charge or a multiplication factor for the admission of a (trauma) patient with ISAR ≥ 2. According to this study, G-DRG B80Z reimbursement should have to be increased by a factor of 1.33 to cover the higher costs in the U65. Selective consideration could also be given to the additional costs incurred primarily due to the higher diagnostic and staffing costs associated with treating older patients.

Clear treatment guidelines are also needed to enable a realistic representation of everyday clinical practice in DRG and lead to fewer losses in deviations from the predefined LOS. The special responsibility of maximum care providers with their additional costs must also be better taken into account.

Providing health care to an increasingly aging society affects almost all specialties and represents one of the greatest personnel and financial challenges for the public health system in the coming decades. Although the calculations in this study are based on German hospital accounting data, the DRG system has become part of hospital financing worldwide. In general, many industrialized nations are confronted with the problems of demographic change and the related conflicts of cost-expenditure imbalance. Therefore, we believe that our exemplary calculations are also transferable internationally.

Further adaptation of the DRG system to the growing costs caused by older patients is imperative. The current situation must not lead to a cost-related reduction in care as a negative consequence of deliberately lower expenses in the area of diagnostics and monitoring. This is neither justifiable in terms of health policy nor ethically. Ensuring the quality of care must be the top priority in the healthcare system. This is currently not sufficiently taken into account in our DRG billing system based on flat rates per case.

### 4.6. Limitations

The major limitation of the work, as mentioned above, is the determination of costs. The cost calculations are simplified and do not take into account the complex rosters and special compensation of medical staff or intangible costs such as the provision of materials and personnel, quality assurance, and maintenance of university structures [28]. These cost items are difficult to track and were not included for the sake of clarity. However, a more in-depth cost calculation would likely result in a higher cost-revenue deficit and support the study’s statement. The exclusion of occupational injuries, particularly in the U65 group, could introduce bias by excluding a potentially large group.

Another limitation is the monocentric study design. The cost-revenue analysis is that of a university hospital. In addition, this also reflects only the urban demographics of a large city.

Another potential source of error is the recording of personnel costs, which are based on an observation period of three months and are included in the study as calculated averages. These factors depend on the situation, the staff, and the patients, so deviations are possible.

## 5. Conclusions

It can be summarized that the inpatient treatment of patients with mTBI does not lead to a cost and proceeds deficit on the balance sheet. However, it has been shown by way of example that in the DGR system a high cost and proceeds discrepancy arises between younger and older patients, with a consequent relevant reimbursement difference. It has been shown that the DRG calculation basis does not always reflect clinical reality. For example, the length of stay targeted from an economic point of view does not correspond to clinical reality. Furthermore, the problem of the DRG system lies in the flat-rate remuneration, which, in view of the demographic developments in Europe, can lead to a considerable discrepancy between DRG reimbursement and coverage of the running costs of a hospital. An additional adjustment of the DRG to the demographic change, p.e. in the form of an additional charge or a multiplication factor will be unavoidable in the future in order to cover the higher costs of treating geriatric patients.

## Figures and Tables

**Table 1:** Sociodemographic and clinical data of patients with mTBI (n = 220) stratified by age U65 (n = 163) and G65 (n = 57).

	DRG B80Z (n = 220)	U65 (n = 163)	G65 (n = 57)	*p*-Value
Sex (male; %)	57.3	60.1	49.1	0.157
Age (y; median (IQR))	49 (32–66)	40 (29–51)	79 (74–86)	
ISAR (pts., median (IQR))			4 (4–5)	
GCS (pts., median (IQR))	15 (14–15)	15 (14–15)	15 (15–15)	0.218
Neurological Symptoms (n, %)	138 (62.7)	125 (76.7)	13 (22.8)	<0.001
Blood alcohol ≥ 0.3‰ (n, %)	41 (18.6%)	34 (20.9%)	7 (12.3%)	<0.001
Oral Anticoagulation (n, %)	62 (28.2)	28 (17.2)	39 (68.4)	<0.001
Relevant disorder (n, %)	31 (14.1)	7 (4.3)	24 (42.1)	0.002
Social care deficit (n, %)	42 (19.1)	23 (14.11)	19 (33.3)	0.19
Inpatient days (total; d)	414	275	139	0.007
Length of stay (d, mean ± SD)	1.9 ± 1.4	1.7 ± 0.8	2.4 ± 1.9	0.007

y = years; IQR = Interquartile range; ISAR = Identification of Seniors At Risk Score; GCS = Glasgow Coma Scale; d = days. Relevant pre-existing disorder, p.e. dementia, neurodegenerative disease, coagulopathy, etc.

**Table 2:** Cost calculation of patient’s admission via ED stratified by age.

		U65 (n = 163)		G65 (n = 57)	
Medical Service	Price (€)	n	Costs (€)	n	Costs (€)
cCT	123.44	38	4690.72	54	6665.76
X-ray extremities	29.63	27	800.01	13	385.19
X-ray spine	12.34	47	579.98	43	530.62
X-ray pelvis	12.34	9	111.06	33	407.22
X-ray thorax	11.52	32	368.64	23	264.96
Routine blood test	22.45	168	3771.60	67	1504.15
BGA	0.37	173	64.01	89	32.93
Physician (Appendix B)	15.38/22.65	163	2506.94	57	1291.05
Nurse (Appendix B)	15.50/20.82	163	2526.50	57	1186.74
Total costs			15,419.46		12,268.62
Ø cost per case			94.60		215.24

cCT= Cranial Computed Tomography; BGA= Blood Gas Analysis. Cost calculation per minute for physician and nurse can be found in Appendix B.

**Table 3:** Cost and proceeds calculation for U65 vs. G65 with DRG B80Z.

	U65 (n = 163)		G65 (n = 57)	
Costs and Proceeds	n/d	€	d	€
*ED (n)*	163	15,419.46	57	12,268.62
*Trauma ward (d)*	214	68,459.61	76	26,999.01
*Monitor bed (d)*	61	20,026.51	63	14,052.24
Total costs	163	103,905.58	57	53,319.87
Ø Costs per patient/case		637.46		935.44
Refund DRG B80Z	163	174,043.69	57	71,704.07
Ø Refund per patient/case		1067.75		1257.97
Cost-proceed difference		70,138.11		18,384.20
Ø Difference per patient/case		430.30		322.53

ED = Emergency Department, d = days, n = number of cases.

## Data Availability

Not applicable.

## Appendix A

Based on the G-DRG B80Z, the reimbursement (Euro [€]) was calculated according to the number of patients/cases (n) in both groups.

Effective Assessment Ratio [EAR] is a measure of the average expense of treatment of an DRG per case. In 2019, the factor for G-DRG B80Z was 0.366. Charges and surcharges due to undercutting (<2 days) or exceeding (≥4 days) of the case-related predefined length of stay were calculated with factor −0.142 and +0.113, respectively [12].

**Table A1:** Reimbursement calculation (G-DRG B80Z) according to age U65 (n = 163) and G65 (n = 57).

			U65		G65	
LOS (d)	EAR	B80Z (€)	n	R (€)	n	R (€)
1	0.224	794.07	74	58,761.18	18	14,293.26
2	0.366	1297.46	74	96,012.04	25	32,436.50
3	0.366	1297.46	11	14,272.06	5	6487.30
4	0.479	1698.04	2	3396.08	3	5094.12
5	0.592	2098.62	1	2098.62	1	2098.62
6	0.705	2499.20	0		1	2499.20
7	0.818	2899.79	1	2899.79	0	
8	0.931	3300.37	0		3	9901.11
9	1.044	3700.95	0		1	3700.95
Reimbursement (total):			163	174,043.69	57	76,511.06
Average per case:			1	1067.75	1	1342.30

LOS = Length of stay; d = days; EAR= Effective Assessment Ratio. R = Reimbursement, € = Euro.

## Appendix B

To calculate personnel costs per minute [min], physicians and nursing staff were considered separately according to their specific remuneration (2019). The individual costs for materials and equipment are recorded individually for each treatment case by the commercial controlling department of the department. For variable, examination-dependent values, an average value was determined using commercial controlling data. Indirect direct costs (infrastructure, laundry, etc.) are included in the inpatient stay costs.

The additional costs of documentation, certification and quality assurance could not be included in these calculations, nor could the costs arise from organization and infrastructure. Indirect or intangible costs, such as the loss of human resource potential, which primarily affects the U65 group, were also not recorded.

### Appendix B.1. Personnel Cost Calculation

The working hours calculation assumes a 365-day year with 30 vacation days and 42 weekly working hours for the physician service [P] and 38.5 weekly working hours for the nursing service [N]. The gross annual hours were used to calculate the net annual hours, considering a nonproductive rate (i.e., sick leave) of 2.1% for P and 6.85% for N [15]. Due to the different pay scales, an average annual salary is calculated for the respective service groups.

Supplements have not been considered for the purposes of clarity. The employer’s contribution for non-wage costs of 19.435% in 2019 includes health, pension, and unemployment insurance as well as the insolvency benefit contribution for an employee of wage tax class 1 and with statutory health insurance.

To determine the working time required by the respective staff group for patient care in the ED and on the trauma ward, the treatment time, including admission, ward rounds, reporting and care, was documented by both staff groups over a period of three months, so that an exemplary average value was determined in each case.

The average annual salary including the employer’s contribution for the attending physician [P] was €80,422.76. The net annual hours were calculated as described in Appendix B. From this, the doctor’s minute rate of €0.649 per min could be calculated. For the nursing staff on the trauma ward [NW], the annual salary was €46,504.44 with a minute rate of €0.429 per minute. Nursing staff in the emergency department [NED] received an annual salary of €48,194.64 and €0.444 per minute.

**Table A2:** Per-minute cost for physician (Germany, 2019).

Physician	Grade (Year)	Annual Salery (€)	Incl. 19.435% (€)
	1	57,253.92	69,735.72
	4	67,737.12	81,579.72
	7	75,148.32	89,952.84
Ø Annual cost:			80,422.76
Annual gross hours	365–114 days (weekends and holidays 2019) = 251 days		
	42 h/ 5 days = 8.4 working h per day 251 days × 8.4 h = 2108.4 working h per year		
Annual net hours	2108.4 h per year–2.1% nonproductive rate = 2064.1 h = 12,3846 min		
Cost per minute	€80,422.76/12,3846 min = 0.649€ per min		

**Table A3:** Per-minute cost for nursing stuff (Germany, 2019).

Nursing Staff	Grade (Year)	Annual Salery (€)	Incl. 19.435% (€)
	3 (NW)	35,669.28	43,607.52
	5 (NW)	40,408.44	49,401.36
	3 (NED)	37,407.36	45,732.48
	5 (NED)	41,435.40	50,656.80
Ø Annual cost NW			46,504.44
Ø Annual cost NED			48,194.64
Annual gross hours	365–114 days (weekends and holidays 2019) = 251 days		
	38.5 h/5 days = 7.7 working h per day 251 days × 7.7 h = 1940.4 working h per year		
Annual net hours	1940.4 h per year–6.85% nonproductive rate = 1807.5 h = 10,8450 min		
Cost per minute NW	€46,504.44/108450 min = €0.429/min		
Cost per minute NED	€48,194.64/108450 min = €0.444/min		

NW = Nurse trauma ward, NED = Nurse Emergency Department.

### Appendix B.2. ED Pesonnel Cost Calculation (per Minute Costs)

The average time required by the medical staff for an admission to ED, including patient’s history, examination, treatment and reporting, amounts to an average of 23.7 min for U65 patients and 31.5 min for G65. The treating nurse has an additional workload due to triage, patient and material preparation, patient transfer and post-processing of the treatment room. This results in costs of €15.38 for physician treatment and €15.50 for nursing treatment for the U65 group. Corresponding to the higher time expenditure, this is 22.65 € for P and 20.82 € for N for G65.

### Appendix B.3. Inpatient Cost Calculation

Since only a few costs can be directly attributed to the inpatient treatment of mTBI observation, flat-rate values for the stay in a trauma ward and for a monitor bed were determined with the help of the commercial controlling data. For a day on the trauma ward, the flat rate in 2019 was €300.00. Added were the costs for the time spent by the physician on daily rounds, examinations and documentation: 13.4 min/d for U65 and 27.8 min/d for G65. Accordingly, medical personnel costs of €8.44/d for U65 and €17.52/d for G65 were incurred on the trauma ward. The NW costs €11.15 (U65) and €33.89 (G65) for the care and observation of the clinical condition of the mTBI patients (26 min/d for U65 and 79 min/d G65).

For an inpatient day on a monitor bed, a flat rate of €512.00 was calculated from the revenue share that the emergency department receives from the respective DRG in accordance with commercial controlling. The costs for nursing care in the emergency department are covered by this flat rate. Medical rounds, including examinations and documentation, take place here at least every two hours; the preparation of the doctor’s letter is also included. This resulted in costs of €43.48/d for U65 (67 min/d) and €49.32/d (76 min/d) for G65.

**Table A4:** Cost of stay per day on trauma ward and monitor bed.

		U65		G65	
	Price (€)	n	Costs (€)	n	Costs (€)
**Trauma ward**					
Flate-rate	300.00	214	64,200.00	76	22,800.00
Routine blood tests	22.45	3	67.35	13	291.85
P	8.44/17.52	214	1806.16	76	1331.52
NW	11.15/33.89	214	2386.10	76	2575.64
Total costs		214	68,459.61	76	26,999.01
Ø Costs per day			319.90		355.25
Ø Cost per patient/case (n = 127/32)			539.05		843.72
**ED**					
Flat-rate	512.00	61	31,232.00	63	12,800.00
Routine blood tests	0.37	89	32.93	62	22.94
P	43.48/49.32	61	2652.28	63	3207.26
Total costs		61	20,026.51	63	14,052.24
Ø Costs per day			328.30		223.05
Ø Cost per patient/case (n = 36/25)			556.29		562.09
